# FRQ-CK1 Interaction Underlies Temperature Compensation of the *Neurospora* Circadian Clock

**DOI:** 10.1128/mBio.01425-21

**Published:** 2021-06-29

**Authors:** Yue Hu, Xiaolan Liu, Qiaojia Lu, Yulin Yang, Qun He, Yi Liu, Xiao Liu

**Affiliations:** a State Key Laboratory of Mycology, Institute of Microbiology, Chinese Academy of Sciences, Beijing, China; b College of Life Sciences, University of the Chinese Academy of Sciences, Beijing, China; c Department of Physiology, University of Texas Southwestern Medical Center, Dallas, Texas, USA; d State Key Laboratory of Agrobiotechnology and MOA Key Laboratory of Soil Microbiology, College of Biological Sciences, China Agricultural University, Beijing, China; University of Georgia

**Keywords:** *Neurospora*, casein kinase 1, circadian clock, protein phosphorylation, temperature compensation

## Abstract

Temperature compensation is a fundamental property of all circadian clocks; temperature compensation results in a relatively constant period length at different physiological temperatures, but its mechanism is unclear. Formation of a stable complex between clock proteins and casein kinase 1 (CK1) is a conserved feature in eukaryotic circadian mechanisms. Here, we show that the FRQ-CK1 interaction and CK1-mediated FRQ phosphorylation, not FRQ stability, are main mechanisms responsible for the circadian temperature compensation phenotypes in *Neurospora*. Inhibition of CK1 kinase activity impaired the temperature compensation profile. Importantly, both the loss of temperature compensation and temperature overcompensation phenotypes of the wild-type and different clock mutant strains can be explained by temperature-dependent alterations of the FRQ-CK1 interaction. Furthermore, mutations that were designed to specifically affect the FRQ-CK1 interaction resulted in impaired temperature compensation of the clock. Together, these results reveal the temperature-compensated FRQ-CK1 interaction, which results in temperature-compensated CK1-mediated FRQ and WC phosphorylation, as a main biochemical process that underlies the mechanism of circadian temperature compensation in *Neurospora*.

## INTRODUCTION

Circadian clocks are critical time-keeping systems that control daily molecular, physiological, and behavior activities in most eukaryotic and prokaryotic organisms (1–4). In addition to the ∼24-h free-running periodicity and ability to be entrained by environmental stimuli, temperature compensation is a fundamental property of all circadian clocks. Temperature compensation ensures that period length remains relatively constant within a physiologically relevant range of temperatures to allow clocks to be functional in different seasons (5). The molecular mechanisms of the circadian clock have been well characterized in different eukaryotic model organisms (6–8). The core eukaryotic circadian oscillators that drive the near 24-h rhythmicity involve autoregulatory transcription-translation-based negative feedback loops. In these negative feedback loops, PAS-domain transcription factors activate the transcription of the clock genes encoding the negative elements, which feed back to repress their own transcription by inhibiting the activity of the transcription factors. In *Neurospora*, *Drosophila*, and mice, FREQUENCY (FRQ), PERIOD (PER), and mPERs, respectively, are the main negative elements in the circadian negative feedback loops.

Despite evolutionary distance, eukaryotic circadian mechanisms share remarkable similarities (6–8). Posttranslational modification of clock proteins by phosphorylation is highly conserved from fungi to mammals. Multiple kinases, including casein kinase 1 (CK1), CK2, and protein kinase A, phosphorylate FRQ in *Neurospora* and PER in insect and mammalian systems to regulate circadian period length and the stability of the negative elements (9–17). Among these kinases, CK1 plays a major role in both FRQ and PER phosphorylation. In addition, CK1 forms a stable stoichiometric complex with FRQ in *Neurospora* and with PER in *Drosophila* and mammals (12, 13, 18–24). This ability of CK1 to interact tightly with FRQ and PER is unlike the typical weak and transient kinase-substrate interactions, suggesting an important conserved function in eukaryotic clock mechanisms. We previously showed that the interaction between FRQ and CK1a in *Neurospora* was the main determinant of circadian period length at room temperature (25).

How circadian clocks achieve temperature-compensated periodicity of approximately 24 h is a fundamental question in chronobiological research. Many models, ranging from opposing and balanced biochemical reactions to an amplitude model, have been proposed to explain the mechanism of temperature compensation (26–29). Consistent with the balancing model, kinases, phosphatases, and different phosphorylation events have been shown to have opposing functions in determining degradation of FRQ and PER, and competing phosphorylation events determine PER2 degradation rate and temperature compensation (13, 14, 30–33). Studies using recombinant CK1 and short synthetic peptides of PER suggest that temperature-compensated phosphorylation events also contribute to temperature compensation mechanisms in mammalian cells (34, 35). In *Neurospora*, CK2 is implicated in temperature compensation, because mutants of CK2 are temperature overcompensated (36). Unlike CK1a, CK2 does not form a stable complex with FRQ in *Neurospora*. Although different kinases have been shown to be involved in temperature compensation, the molecular mechanism of how these kinases regulate temperature compensation is still not clear.

In the *Neurospora* core circadian negative feedback loop, FRQ and its partner, FRQ-interacting RNA helicase (FRH), are the negative elements (6, 37). The two PAS-domain WHITE COLLAR proteins, WC-1 and WC-2, form a complex, WCC, that binds to the C-box of the *frq* promoter to activate transcription of *frq* (38–40). To close the negative feedback loop, FRQ-FRH inhibits the activity of the WCC by promoting WC phosphorylation by CK1 and CK2 (24, 41–44). The stable interaction between FRQ and CK1a is mediated by the FCD1 and FCD2 interaction domains on FRQ (24, 45, 46). Importantly, deletion or mutations of either FCD can completely abolish the stable FRQ-CK1a interaction and CK1a-mediated FRQ phosphorylation *in vivo*, indicating that FRQ and CK1a need to form a stable complex before FRQ can be phosphorylated by CK1a at different sites. Over time, FRQ is progressively phosphorylated at about 100 phosphorylation sites in wild-type *Neurospora* strains (13, 14, 24, 47–50). Phosphorylation of FRQ plays a major role in determining circadian periodicity (13, 14, 17, 25, 51).

Due to the role of phosphorylation in regulating protein stability, the degradation of clock proteins was previously thought to be a major determinant of circadian period length and was also proposed to be the main process that determines temperature compensation (17, 30, 36, 52–57). However, we and others previously showed that FRQ phosphorylation, but not its degradation rate, is critical in period determination in *Neurospora* at room temperature (25, 51). Our work further suggests that the FRQ-CK1 interaction is the main rate-limiting process in circadian period determination at room temperature due to its dual roles in both arms of the circadian negative feedback loop (25). The FRQ-CK1 interaction determines the FRQ phosphorylation profile, which feeds back to either promote or reduce the interaction itself. It also determines the efficiency of the circadian negative feedback process by mediating FRQ-dependent WC phosphorylation.

In this study, we investigated the mechanism of temperature compensation in *Neurospora* by examining the wild-type and clock mutant strains under conditions when temperature compensation was impaired. Our results showed that inhibited CK1 activity or impaired FRQ-CK1a interaction could change the temperature compensation profile of the clock. Moreover, we demonstrated that the maintenance of FRQ-CK1a interaction, but not CK1a protein level or kinase activity at different temperatures, correlates with the temperature compensation phenotypes of different mutants in *Neurospora*, suggesting that temperature-compensated FRQ-CK1a interaction is the main mechanism responsible for temperature compensation of the clock. Together, these results established the FRQ-CK1a interaction as the main biochemical process that defines temperature compensation in the *Neurospora* clock.

## RESULTS

### Alteration in FRQ stability is not responsible for impaired temperature compensation profile in the wild-type and clock mutant strains.

**FIG 1:**
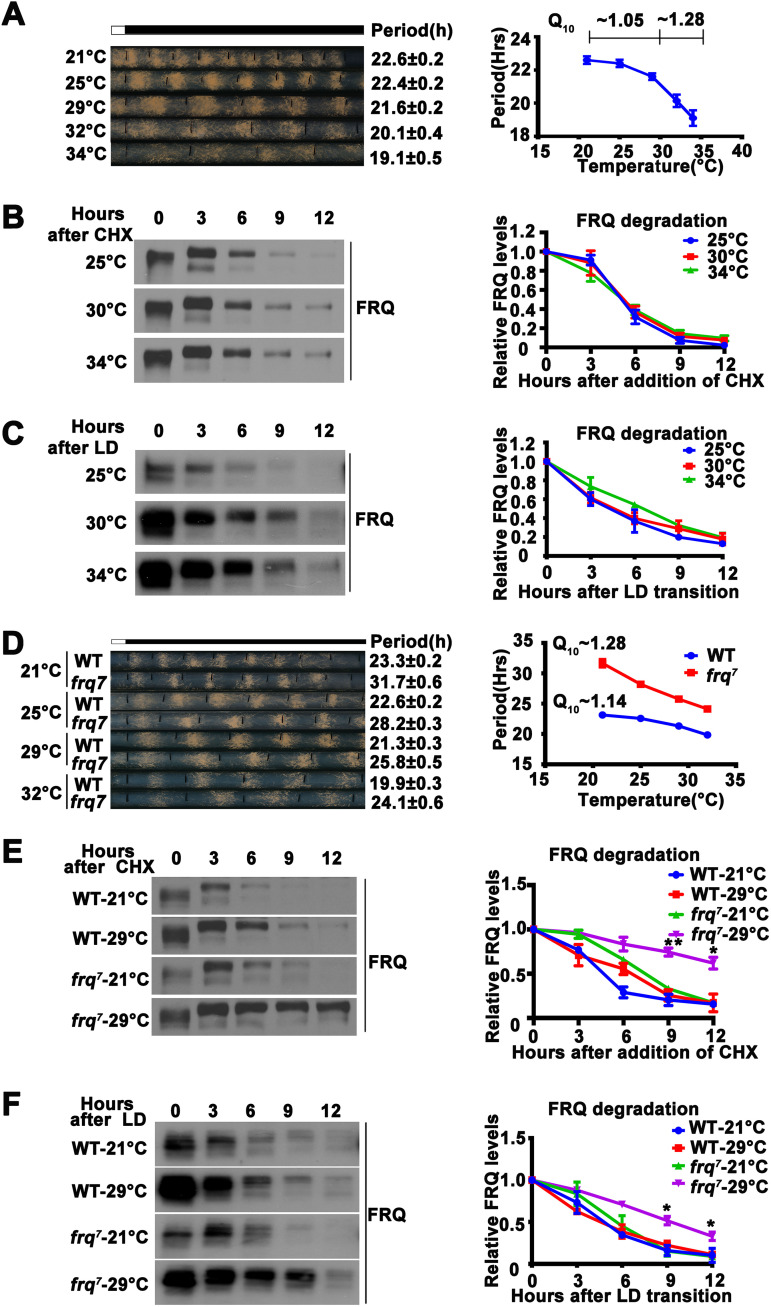
Altered FRQ stability is not responsible for temperature compensation. (A, left) Representative photographs of race tubes used to evaluate circadian conidiation rhythm of the wild-type strain at indicated temperatures. Periods are given to the right. (Right) Plot of period versus temperature. Error bars are standard errors of means (*n* = 5). (B, left) Image of Western blot for FRQ from the wild-type strain grown in constant light at the indicated temperatures for the indicated number of hours after CHX addition. (Right) Plot of relative FRQ level as a function of time after CHX addition over a range of temperatures. Error bars are standard deviations (*n* = 3). The levels of large and small forms of FRQ due to alternative splicing and their various phosphorylated species were used for quantification. (C, left) Image of Western blot for FRQ from the wild-type strain grown in constant light for one day, transferred into constant darkness, and harvested at the indicated time. (Right) Plot of relative FRQ level as a function of time after light/dark (LD) transition over a range of temperatures. Error bars are standard deviations (*n* = 3). (D, left) Representative photographs of race tubes used to evaluate circadian conidiation rhythms of the wild-type (WT) and *frq^7^* strains at the indicated temperatures. Periods are given to the right. (Right) Plot of period versus temperature. Error bars are standard errors of means (*n* = 5). (E, left) Image of Western blot for FRQ from wild-type and *frq^7^* strains grown in constant light at 21 and 29°C for the indicated number of hours after CHX addition. (Right) Plot of relative FRQ quantity as a function of time after CHX addition over a range of temperatures. Error bars are standard deviations (*n* = 3). (F, left) Image of Western blot for FRQ from wild-type and *frq^7^* strains grown at 21 and 29°C for the indicated number of hours after LD transition. (Right) Plot of relative FRQ quantity as a function of time after LD transition over a range of temperatures. Error bars are standard deviations (*n* = 3). *, *P* < 0.05; **, *P* < 0.01; Student’s *t* test.

The circadian period of wild-type *Neurospora* is temperature compensated between 20°C and 30°C, with a temperature coefficient (Q_10_) of approximately 1.05 (58) (Fig. 1A). Above 30°C, however, temperature compensation is partially lost, and the period becomes markedly shorter as temperature increases (∼19 h at 34°C), with a Q_10_ value of about 1.28 (Fig. 1A) (58). To understand the mechanism of temperature compensation, we first examined whether a change in FRQ stability was responsible for the impaired temperature compensation in the wild-type *Neurospora* strain above 30°C. Comparison of the FRQ degradation rates at 25, 30, and 34°C after the addition of protein biosynthesis inhibitor cycloheximide (CHX) showed that FRQ stability was not significantly different at these temperatures (Fig. 1B). To exclude the effect of CHX at different temperatures, we measured the degradation rate of FRQ following a light-to-dark (LD) transition and found the stability of FRQ was also similar at different temperatures (Fig. 1C). This result indicated that the partial loss of temperature compensation in the wild-type strain above 30°C cannot be explained by a change in FRQ stability.

The long period mutant *frq^7^* is a *Neurospora* clock mutant that has a partial loss of temperature compensation between 20 to 30°C (58, 59). As temperature was increased, the period of *frq^7^* shortened more rapidly than the period of the wild-type strain (Fig. 1D). To determine whether the FRQ stability correlated with the temperature compensation defect of *frq^7^*, we compared FRQ degradation rates at 21°C and 29°C. As expected, FRQ stability was similar at both temperatures for the wild-type strain (Fig. 1E). In *frq^7^*, however, FRQ was more stable at 29°C than at 21°C (Fig. 1E). Similar degradation profiles were also observed after an LD transition (Fig. 1F). Since increased stability of FRQ is normally correlated with period lengthening, this is opposite to what is predicted if a change of FRQ stability is responsible for its period shortening at high temperatures. Together, these results indicated that maintenance of FRQ stability is not the mechanism underlying temperature compensation in *Neurospora*.

### CK1 is involved in regulating temperature compensation.

**FIG 2:**
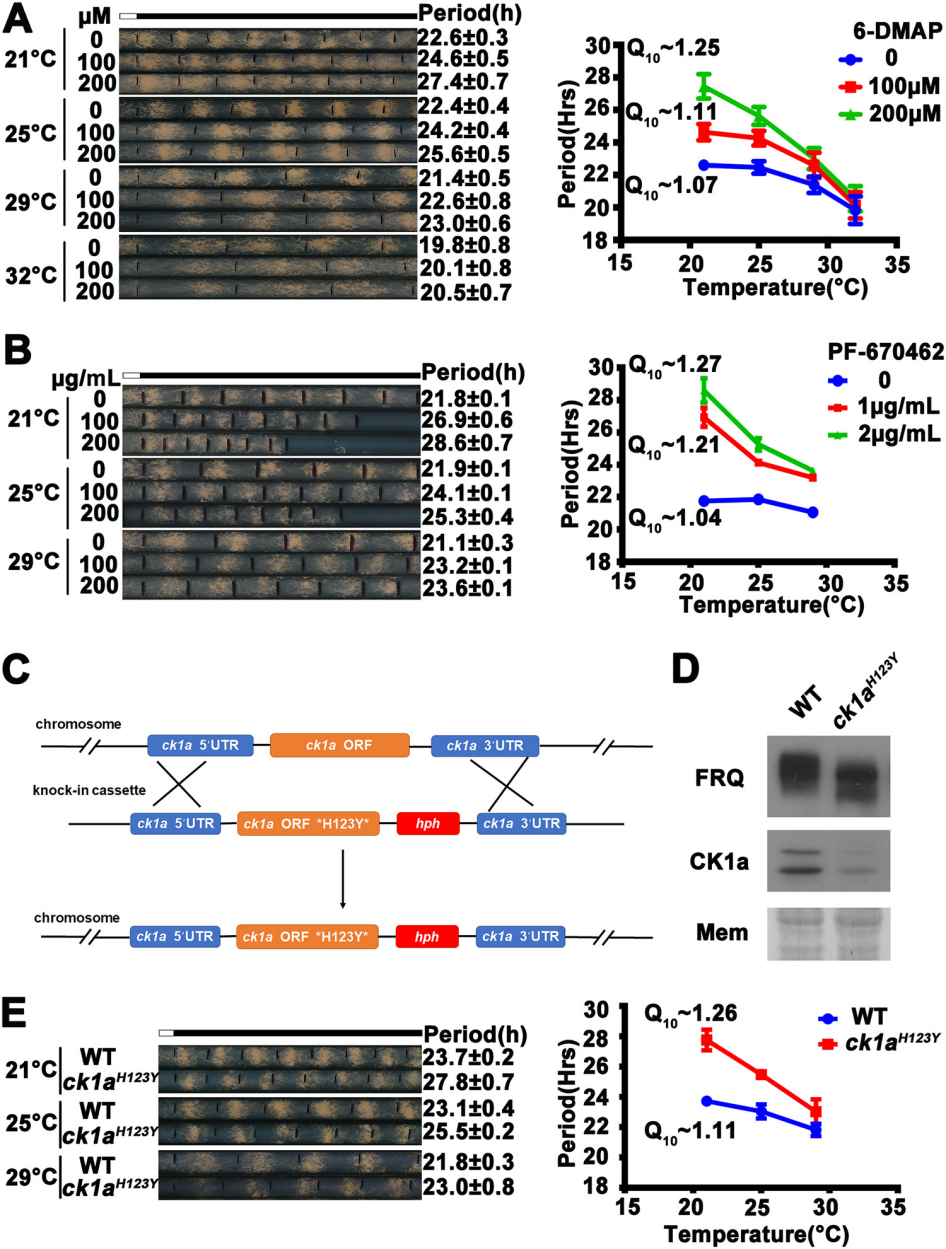
Casein kinase 1 is involved in temperature compensation of the *Neurospora* clock. (A, left) Representative photos of race tubes used to evaluate effects of 6-DMAP treatment on conidiation rhythms in the wild-type strain over a range of temperatures. (Right) Plot of period versus temperature at indicated 6-DMAP concentrations. Error bars are standard errors of means (*n* = 3). (B, left) Representative photos of race tubes used to evaluate effects of PF670462 treatment on conidiation rhythms of the wild-type strain. (Right) Plot of period versus temperature at indicated PF670462 concentrations. Error bars are standard errors of means (*n* = 3). (C) Diagram depicting the strategy for creation of the homokaryotic *ck1a^H123Y^* strain by homologous recombination (see Materials and Methods for details). (D) Western blot of FRQ and CK1a protein levels in the wild-type and *ck1a^H123Y^* strains. (E, left) Representative photos of race tubes used to evaluate conidiation rhythms of the WT (*ck1a^WT^*) and *ck1a^H123Y^* mutant. (Right) Plot of period versus temperature for wild-type and mutant strains. Error bars indicate standard errors of means (*n* = 3).

Because of the critical role of FRQ phosphorylation in regulating circadian period length in *Neurospora*, we examined whether the inhibition of FRQ phosphorylation affects temperature compensation. The general kinase inhibitor 6-DMAP has been shown to inhibit FRQ phosphorylation and lengthen the circadian period *in vivo* (17). As expected, 6-DMAP treatment lengthened the period of the conidiation rhythms of the wild-type strain at 25°C in a dose-dependent manner (Fig. 2A). However, as temperature increased from 21°C to 32°C, the effect of 6-DMAP treatment on period length decreased, and 6-DMAP had only a very modest effect on period length at 32°C. Q10 (21 to 30°C) increased from approximately 1.07 in the absence of 6-DMAP to approximately 1.25 at 200 μM 6-DMAP, indicating that the 6-DMAP treatment resulted in a partial loss of temperature compensation in a dose-dependent manner.

Since CK1a (encoded by NCU00685) is the major kinase that phosphorylates FRQ, we examined the involvement of CK1a in temperature compensation. PF-670462 was a CK1-specific inhibitor that was previously shown to inhibit FRQ phosphorylation (45, 60). As expected, PF-670462 treatment resulted in hypophosphorylation of FRQ in the wild-type strain and period lengthening (Fig. 2B; see also Fig. S1A in the supplemental material). Similar to 6-DMAP treatment, PF-670462 treatment also resulted in partial loss of temperature compensation of the circadian conidiation rhythms (Fig. 2B), suggesting that CK1 activity is critical for temperature compensation.

*ck1a* is an essential gene in *Neurospora* (47). Although we previously created a *ck1a^L^* (M83I) knock-in mutant that exhibited a long-period circadian phenotype (24), growth and development of this mutant were severely impaired, making it difficult to examine its circadian phenotypes at different temperatures. To obtain genetic evidence of the involvement of CK1a in temperature compensation, we generated another *ck1a* knock-in mutant in which a conserved histidine residue at position 123 is mutated to tyrosine (Fig. 2C). The same mutation in the budding yeast CK1 homolog YCK2 was previously shown to regulate CK1 activity (61), and a similar mutation in *Drosophila doubletime* (*dbt*) resulted in arrhythmicity or long circadian period (62). In *Neurospora*, the CK1a H123Y mutation led to hypophosphorylation of FRQ and decreased CK1a protein levels (Fig. 2D). The decreased CK1a^H123Y^ protein levels were not caused by degradation, indicating H123Y mutation affects protein folding of CK1a (Fig. S1B) or posttranscriptional regulation of CK1a (63). As expected, the *ck1a^H123Y^* mutant exhibited a long period at 25°C (Fig. 2E). Comparison of the period lengths at different temperatures showed that the Q_10_ value of the *ck1a^H123Y^* strain was increased to 1.26, indicating a partial loss of temperature compensation (Fig. 2E). Together, these results indicated that CK1a is involved in regulating the mechanism of temperature compensation.

10.1128/mBio.01425-21.1 FIG S1 FRQ phosphorylation by CK1 is involved in temperature compensation. (A) Western blot analyses of hypophosphorylation of FRQ in the wild-type strain after treatment with the indicated concentrations of PF670462. (B, left) Western blot analyses of CK1a in the wild-type and *ck1a^H123Y^* strains after CHX treatment. (Right) Plot of relative CK1a levels at indicated hours after CHX treatment. Error bars are standard deviations (*n* = 3). Download FIG S1, TIF file, 2.1 MB. Copyright © 2021 Hu et al. 2021 Hu et al. https://creativecommons.org/licenses/by/4.0/ This content is distributed under the terms of the Creative Commons Attribution 4.0 International license.

### Temperature-sensitive FRQ-CK1a interaction, but not changes in CK1a protein level and its kinase activity, correlates with temperature compensation phenotypes.

**FIG 3:**
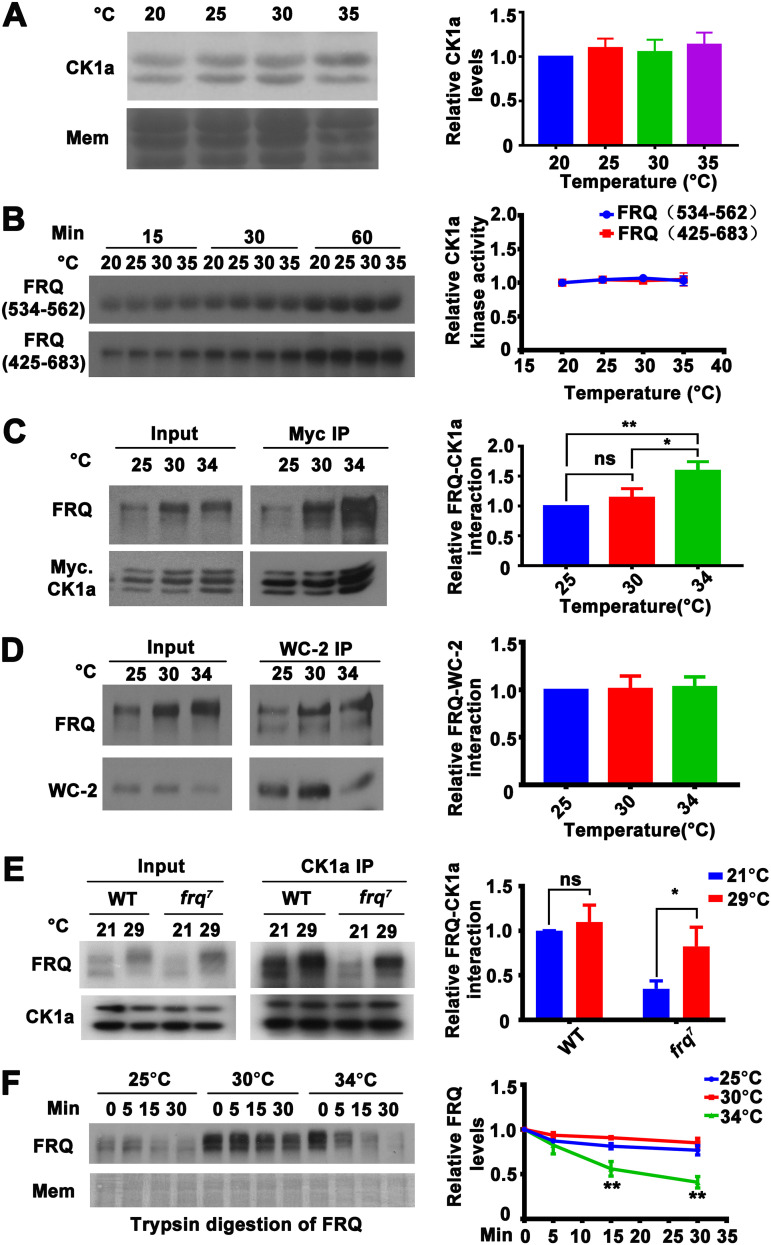
Temperature sensitivity of the FRQ-CK1a interaction explains the temperature compensation profiles of the wild-type and *frq^7^* strains. (A, left) Western blot analysis for CK1a and stained membrane (MEM) (loading control) in extracts of the wild-type strain grown at the indicated temperatures. (Right) Plot of the relative CK1a levels as a function of temperature. Error bars are standard deviations (*n* = 5). (B) *In vitro* kinase assay showing the phosphorylation of GST-FRQ (534-562) and GST-FRQ (425-683) by recombinant His-CK1a at different temperatures. Error bars are standard deviations (*n* = 3). (C, left) Western blot analysis for FRQ precipitated with Myc-CK1a from extracts of *Neurospora* cultures grown in constant light at the indicated temperatures in the presence of quinic acid (QA). (Right) Plot of the relative amount of FRQ-CK1a complex as a function of temperature. Quantification of relative FRQ-CK1a interaction levels is based on the ratio of IP to input and normalized to CK1a level. Error bars are standard deviations (*n* = 3). ns, not significant; *, *P* < 0.05; **, *P* < 0.01; Student’s *t* test. (D, left) Western blot analysis of WC-2 immunoprecipitates from the wild-type strain grown at the indicated temperatures and analyzed for FRQ and WC-2. (Right) Relative amounts of FRQ precipitated with WC-2 as a function of growth temperature (*n* = 6). Quantification of relative FRQ-WC-2 interaction levels is based on the ratio of IP to input and normalized with WC-2 level. (E, left) Western blot analysis for FRQ precipitated with endogenous CK1a from extracts of the wild-type and *frq^7^* strains grown in constant light at 21 and 29°C. (Right) Relative amount of FRQ-CK1a complex at 21 and 29°C. Quantification of relative FRQ-CK1a interaction levels is based on the ratio of IP to input and normalized with CK1a level. Error bars are standard deviations (*n* = 5). ns, not significant; *, *P* < 0.05; Student’s *t* test. (F) Western blot analysis of FRQ from extracts of the wild-type strain grown in constant light at the indicated temperatures and subjected to partial trypsin digestion. Error bars are standard deviations (*n* = 4). **, *P* < 0.01; Student’s *t* test.

To investigate how CK1a regulates temperature compensation, we examined whether CK1a protein levels or kinase activity are temperature sensitive above 30°C when temperature compensation of the wild-type strain becomes impaired. The endogenous CK1a (two alternatively spliced CK1a isoforms) levels did not change significantly from 20°C to 35°C (Fig. 3A), indicating that a temperature-dependent change in CK1a protein levels is not the cause of the partial loss of temperature compensation above 30°C. To determine the biochemical activity of CK1a, we expressed the recombinant full-length *Neurospora* CK1a in Escherichia coli and examined the kinase activity of the purified protein in an assay with two glutathione *S*-transferase (GST)-tagged recombinant FRQ fragments, FRQ (534-562) and FRQ (425-683), as the substrate at different temperatures. CK1a activity toward FRQ peptides *in vitro* was relatively temperature insensitive, and there was no significant difference in kinase activity between 30 and 35°C (Fig. 3B). This result suggested that CK1a kinase activity is relatively insensitive to temperature changes; therefore, changes in kinase activity as a function of temperature alone do not explain the partial loss of temperature compensation above 30°C in the wild-type strain. Interestingly, it was previously reported that the mammalian CK1ε/δ activity toward short peptides is also relatively temperature insensitive *in vitro* (34).

Because the FRQ-CK1a interaction was the major determinant of FRQ phosphorylation and circadian period length at room temperature and the relative level of this interaction was negatively correlated with period length (25), we examined whether the FRQ-CK1a interaction was sensitive to temperature above 30°C in the wild-type strain. A construct for expression of Myc-CK1a was transformed into *Neurospora* to allow the relative interaction between FRQ and CK1a to be monitored by immunoprecipitation (IP) assays using a c-Myc monoclonal antibody (25). Although the relative level of FRQ-CK1a complex (ratio of CK1a-immunoprecipitated FRQ level to the input FRQ level that is normalized with the CK1a level) was similar between 25°C and 30°C, it was significantly increased at 34°C (Fig. 3C). In contrast, the amount of FRQ-WC2 complex was not affected by temperature (Fig. 3D). To further confirm this result, we performed an *in vitro* pulldown assay by incubating the recombinant Flag-tagged CK1a with extracts of wild-type *Neurospora* grown at different temperatures. As expected, relatively more FRQ was precipitated with Flag-CK1a from extracts of *Neurospora* grown at 34°C than at 25°C and 30°C (Fig. S2). Because an increase of the relative FRQ-CK1a interaction resulted in period shortening (25), these findings suggested that at temperature above 30°C, FRQ-CK1a interaction was specifically increased without affecting the FRQ-WC interaction, resulting in enhanced WC complex phosphorylation and inhibition and leading to a shorter period. Therefore, the increase in FRQ-CK1a interaction should be responsible for the partial loss of temperature compensation of the wild-type strain above 30°C.

10.1128/mBio.01425-21.2 FIG S2 FRQ-CK1a interaction is enhanced at high temperature. (Left) Western blot analysis of *in vitro* pulldown assay showing the amount of FRQ precipitated with recombinant Flag-CK1a from extracts of *Neurospora* cultures grown in constant light at the indicated temperatures. (Right) Relative amount of FRQ-CK1a complex at indicated temperatures. Quantification of relative FRQ-CK1a interaction levels is based on the ratio of pulldown to input and normalized to CK1a level. Error bars are standard deviations (*n* = 5). ns, not significant; **, *P* < 0.01; Student’s *t* test. Download FIG S2, TIF file, 1.8 MB. Copyright © 2021 Hu et al. 2021 Hu et al. https://creativecommons.org/licenses/by/4.0/ This content is distributed under the terms of the Creative Commons Attribution 4.0 International license.

If the FRQ-CK1a interaction plays a major role in temperature compensation, it should also explain the temperature compensation defects in clock mutants with impaired temperature compensation. Thus, we examined the FRQ-CK1a interaction in the *frq^7^* strain at different temperatures. The CK1a immunoprecipitation assay showed that the relative amount of FRQ precipitated was increased at 29°C compared to 21°C in the *frq^7^* strain but not in the wild-type strain (Fig. 3E). This is consistent with the partial loss of temperature compensation in the *frq^7^* strain (Fig. 1C). Thus, the altered FRQ-CK1a interaction also explains the partial loss of temperature compensation of the *frq^7^* strain.

We hypothesized that a temperature increase above 30°C induces structural changes in FRQ that affect the FRQ-CK1a interaction in the wild-type strain. To test this possibility, we used a limited trypsin digestion assay performed at 30°C to probe the structure of FRQ in *Neurospora* extracts harvested from the wild-type strain grown at different temperatures in constant light. In extracts from the wild-type culture grown at 34°C, FRQ was significantly more sensitive to trypsin digestion than FRQ from culture grown at 30°C (Fig. 3F), suggesting that a change in the structural conformation of FRQ above 30°C affects FRQ-CK1a interaction.

### Temperature-dependent FRQ-CK1a interaction also explains the temperature overcompensation phenotype of the *ck2* and FRQ CK2 phosphorylation sites mutants.

**FIG 4:**
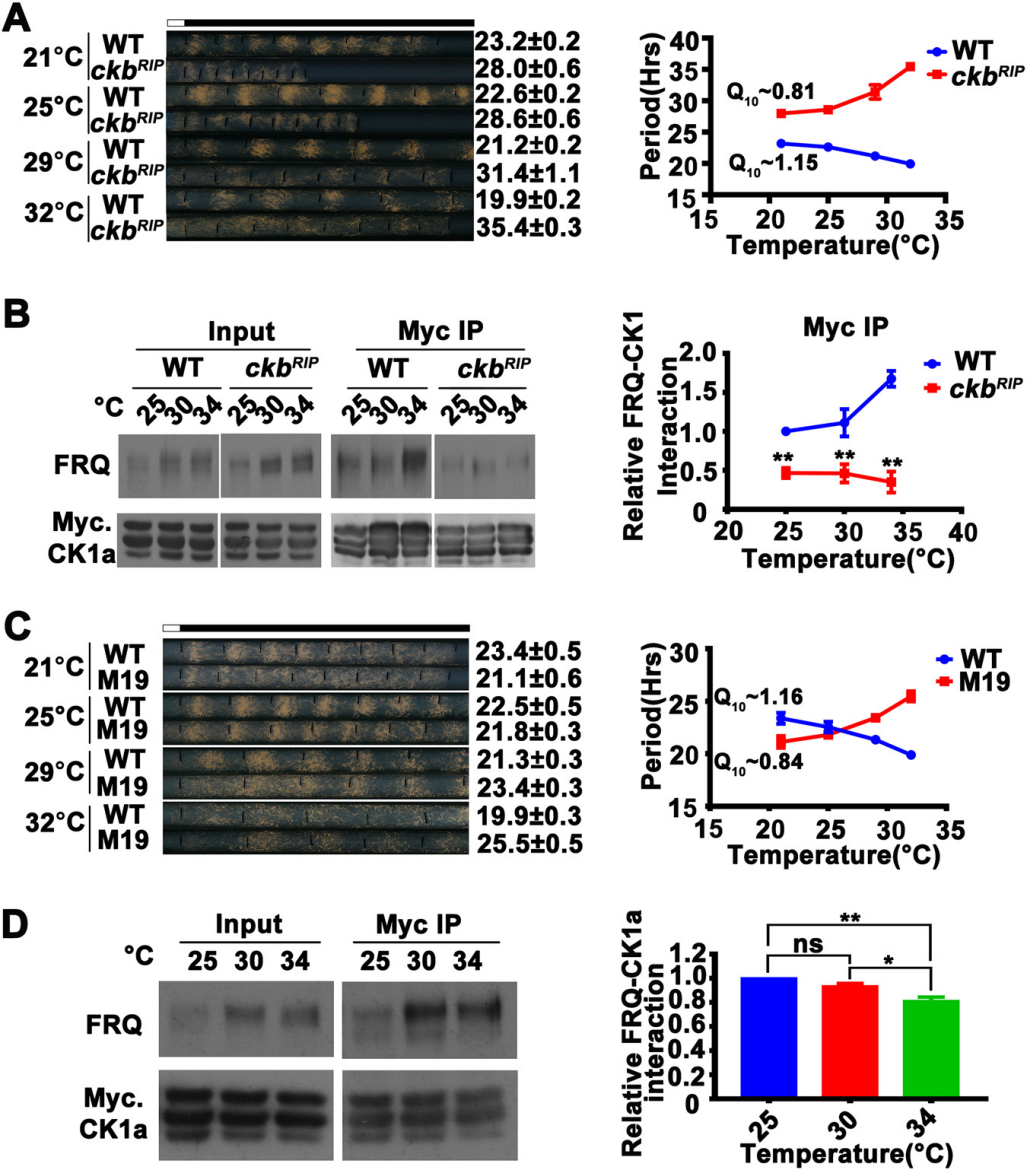
Temperature sensitivity of the FRQ-CK1a interaction results in the temperature overcompensation phenotype of the *ckb^RIP^* strain and CK2 phosphorylation sites of FRQ mutants. (A, left) Representative photos of race tubes used to evaluate conidiation rhythms of the *ckb^RIP^* strain. (Right) Plot of period versus temperature for wild-type and *ckb^RIP^* strains. Error bars are standard errors of means (*n* = 5). (B, left) Western blot analysis for FRQ precipitated with Myc-CK1a from extracts of the wild-type strain and the *ckb^RIP^* strain that expresses Myc-CK1a grown in constant light at the indicated temperatures in the presence of quinic acid. (Right) Plot of the relative amount of FRQ-CK1a complex as a function of temperature. Quantification of relative FRQ-CK1a interaction levels is based on the ratio of IP to input and normalized with CK1a level. Error bars are standard deviations (*n* = 4). **, *P* < 0.01; Student’s *t* test. (C, left) Representative photos of race tubes used to evaluate conidiation rhythms of the wild-type (*frq* complementation strain) and M19 strains. (Right) Plot of period versus temperature for wild-type and M19 strains. Error bars are standard errors of means (*n* = 4). (D) Myc-CK1a immunoprecipitation assays showing that the FRQ-CK1a interaction is temperature overcompensated in the M19 strain that expresses Myc-CK1a grown in constant light at the indicated temperatures. Quantification of relative FRQ-CK1a interaction levels is based on the ratio of IP to input and normalized with CK1a level. Error bars are standard deviations (*n* = 3). *, *P* < 0.05; **, *P* < 0.01; Student’s *t* test.

CK2 was previously shown to be a regulator of temperature compensation in *Neurospora* (36). In contrast to the wild-type strain, *ck2* mutants are temperature overcompensated, meaning that the period lengthens as temperature increases. Although CK2 is also an FRQ kinase, it does not form a stable complex with FRQ (13, 19, 48, 49). Consistent with previous results (36), we observed that the period length of the *ck2* mutant *ckb^RIP^* (48) became longer as the temperature increased (Fig. 4A). We compared the relative amounts of FRQ associated with Myc-CK1a at different temperatures in the wild-type and *ckb^RIP^* strains. FRQ was hypophosphorylated in the *ckb^RIP^* strain at 34°C in constant light. In contrast to the significant increase in FRQ precipitated with CK1a at 34°C relative to 25°C observed in the wild-type strain, their relative association was reduced at the higher temperature in the *ckb^RIP^* mutant (Fig. 4B). In addition, the relative level of FRQ-CK1a interaction was lower in the *ckb^RIP^* strain at all temperatures compared to the wild-type strain, consistent with its long-period phenotype. Thus, alterations of the level of the FRQ-CK1a interaction also explain the temperature overcompensation phenotype of the *ckb^RIP^* mutant.

The effect of CK2 on temperature compensation suggested that CK2-dependent FRQ phosphorylation caused a temperature-dependent decrease of FRQ-CK1a interaction and an increased period length at higher temperatures. To confirm this, we screened FRQ phosphorylation site mutants for temperature overcompensation phenotype. The *frq* M19 mutant has mutations in several phosphorylation sites that match the CK2 consensus sequence (Fig. S3A) (14). Similar to the *ckb^RIP^* strain, the circadian conidiation rhythms of M19 also exhibited a temperature overcompensation phenotype (Q_10_ of 0.84), and its period was increased from 21.1 h at 21°C to 25.5 h at 32°C (Fig. 4C). As predicted, the relative level of the FRQ-CK1a association in extracts of the M19 mutant was significantly decreased at 34°C compared to lower temperatures (Fig. 4D).

10.1128/mBio.01425-21.3 FIG S3 Phosphorylation of FRQ-M19 peptides by CK2a *in vitro*. (A) Amino acid sequences of GST-FRQ (972-990) and GST-FRQ (972-990)-M19. (B) Blot of *in vitro* kinase reactions with purified recombinant His-CK1a and His-CK2a as the kinases and GST-FRQ (972-990) as the substrate. (C) Blot of *in vitro* kinase reactions with purified recombinant His-CK2a as the kinase and GST-FRQ (972-990) and GST-FRQ (972-990)-M19 as the substrates. Download FIG S3, TIF file, 1.5 MB. Copyright © 2021 Hu et al. 2021 Hu et al. https://creativecommons.org/licenses/by/4.0/ This content is distributed under the terms of the Creative Commons Attribution 4.0 International license.

To confirm the sites in FRQ mutated in M19 were phosphorylated by CK2 and not by CK1a, we performed *in vitro* phosphorylation assays using recombinant *Neurospora* CK1a or CK2 with purified GST-fusion peptides containing the wild-type region or the M19 region (Fig. S3A). The wild-type GST-fusion peptide was readily phosphorylated by CK2 but not by CK1a; the peptides containing the M19 mutations were not phosphorylated by CK2 (Fig. S3B and C). These experiments indicate that these sites are indeed CK2 sites. Together, these results indicate that some of the CK2-dependent FRQ phosphorylation causes a decrease in the FRQ-CK1a interaction as temperature increases, resulting in the temperature overcompensation phenotype in the *ckb^RIP^* and M19 strains. Therefore, altered FRQ-CK1a interactions can explain both the loss of temperature compensation and the temperature overcompensation phenotypes of the different clock mutants.

### Mutations that specifically alter the FRQ-CK1a interaction impair temperature compensation.

**FIG 5:**
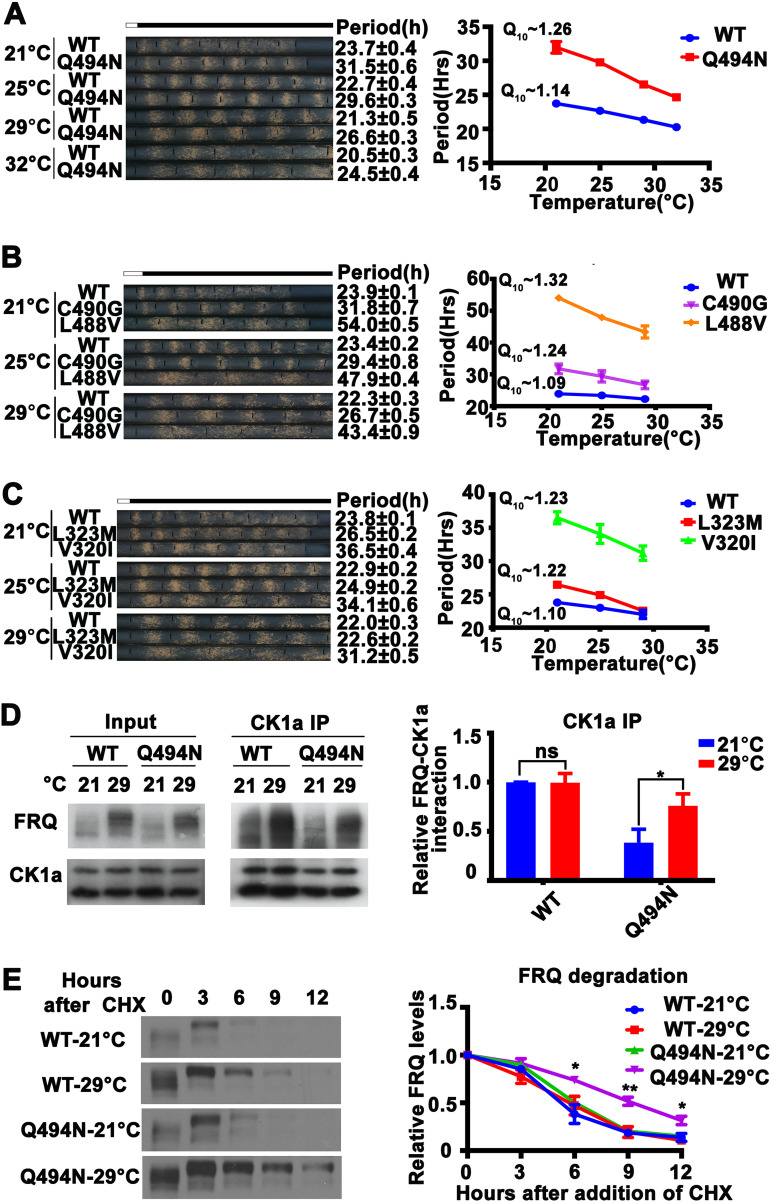
Mutations of the FCD domains of FRQ cause temperature-sensitive FRQ-CK1a association and partial loss of temperature compensation. (A to C, left) Representative photos of race tubes used to evaluate conidiation rhythms of the wild-type (*frq* complementation strain) and FCD mutant strains. (Right) Plot of period versus temperature for wild-type and FCD mutant strains. Error bars are standard errors of means (*n* = 5 to 9). (D, left) Western blot analyses of FRQ immunoprecipitated with anti-CK1a antibody from extracts of the wild-type and Q494N strains grown in constant light at 21 and 29°C. (Right) Plot of relative amount of FRQ in wild-type and Q494N strains. Quantification of relative FRQ-CK1a interaction levels is based on the ratio of IP to input and normalized to CK1a level. Error bars are standard deviations (*n* = 3). ns, not significant; *, *P* < 0.05; Student’s *t* test. (E, left) Western blot analyses of FRQ in the wild-type and Q494N strains grown in constant light at 21 or 29°C for the indicated number of hours after CHX addition. (Right) Plot of relative FRQ levels at the indicated hours after CHX treatment. Error bars are standard deviations (*n* = 3). *, *P* < 0.05; **, *P* < 0.01; Student’s *t* test.

Since our data suggested that the FRQ-CK1a interaction is a major factor that determines temperature compensation, we hypothesized that mutation of the FRQ-CK1a interaction domain would change the temperature compensation profile. To test this, we used *Neurospora* strains with mutations within the FCD1 and FCD2 domains of FRQ that have been previously shown to weaken but not abolish the interaction between FRQ and CK1a, resulting in long-period phenotypes (25). We examined the temperature dependence of circadian conidiation rhythms of mutants with single-amino-acid substitutions in either FRQ FCD1 (L323M and V320I) or FCD2 (L488V, C490G, and Q494N). As expected, all of these FCD mutants displayed partial loss of temperature compensation (Q_10_ value ranging from 1.22 to 1.32) as their period length shortened more rapidly as temperature increased than the wild-type strain (Fig. 5A to C).

To confirm that the temperature compensation phenotypes of the FCD mutants are indeed due to the alterations in the stability of the FRQ-CK1a complex, we examined the FRQ-CK1a interaction in the Q494N mutant by immunoprecipitating the endogenous CK1a. As expected, the relative amount of the FRQ-CK1a complex in the Q494N mutant was significantly higher at 29°C than at 21°C (Fig. 5D). On the other hand, FRQ became more stable at 29°C than at 21°C in the Q494N mutant following either CHX treatment (Fig. 5E) or LD transition (Fig. S4A), further confirming that temperature-dependent changes in FRQ stability do not mediate temperature compensation. Together, these results strongly suggest that the maintenance of FRQ-CK1a interaction underlies the molecular basis of circadian temperature compensation in *Neurospora*.

10.1128/mBio.01425-21.4 FIG S4 Altered FRQ stability is not responsible for temperature compensation in FRQ mutants. (A, left) Image of Western blot for FRQ from the wild-type and Q494N strains grown in constant light at 21 or 29°C for one day, transferred into constant darkness, and harvested at the indicated time. (Right) Plot of relative FRQ level as a function of time after light/dark (LD) transition over a range of temperatures. Error bars are standard deviations (*n* = 3). (B, left) Image of Western blot for FRQ from the wild-type, M9, and M10 strains grown in constant light at 21 or 29°C for one day, transferred into constant darkness, and harvested at the indicated time. (Right) Plot of relative FRQ level as a function of time after light/dark (LD) transition over a range of temperatures. Error bars are standard deviations (*n* = 3). *, *P* < 0.05; Student’s *t* test. Download FIG S4, TIF file, 2.9 MB. Copyright © 2021 Hu et al. 2021 Hu et al. https://creativecommons.org/licenses/by/4.0/ This content is distributed under the terms of the Creative Commons Attribution 4.0 International license.

### Mutations of CK1a phosphorylation sites on FRQ impair temperature compensation.

**FIG 6:**
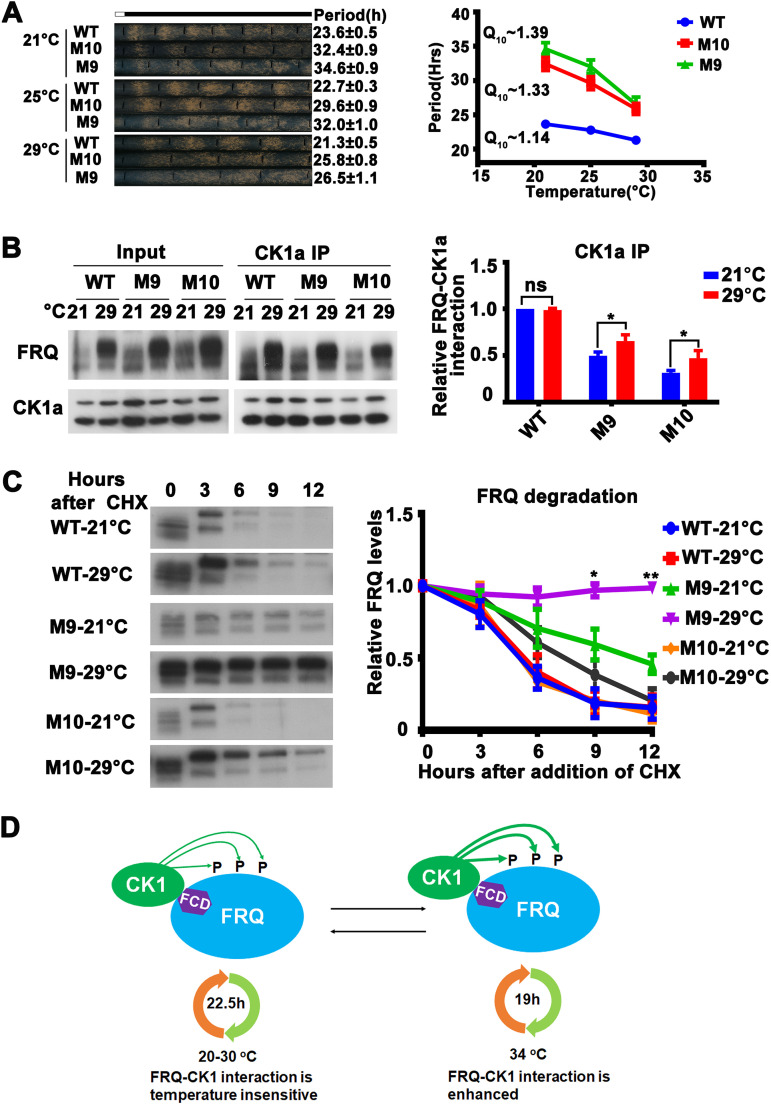
Mutations of CK1a phosphorylation sites on FRQ cause temperature-sensitive FRQ-CK1a interaction and partial loss of temperature compensation. (A, left) Representative photos of race tubes used to evaluate conidiation rhythms of wild-type (*frq* complementation strain) and M10 and M9 mutant strains at the indicated temperatures. (Right) Plot of period versus temperature for wild-type and mutant strains. Error bars indicate standard errors of means (*n* = 5). (B, left) Western blot analyses of FRQ immunoprecipitated with anti-CK1a antibody from extracts of the wild-type, M9, and M10 mutant strains grown in constant light at 21 and 29°C. (Right) Plot of relative amount of FRQ in wild-type, M9, and M10 mutant strains. Quantification of relative FRQ-CK1a interaction levels is based on the ratio of IP to input and normalized to CK1a level. Error bars are standard deviations (*n* = 3). ns, not significant; *, *P* < 0.05; Student’s *t* test. (C, left) Western blot analyses of FRQ in the wild-type, M9, and M10 mutant strains grown in constant light at 21 or 29°C for the indicated number of hours after CHX addition. (Right) Plot of relative FRQ levels at the indicated hours after CHX treatment. Error bars are standard deviations (*n* = 3). *, *P* < 0.05; **, *P* < 0.01; Student’s *t* test. (D) FRQ-CK1a interaction is important for the maintenance of circadian period of the wild-type strain within the physiological temperature range (20 to 30°C). When temperature is above 34°C, the FRQ-CK1a interaction is enhanced, resulting in period shortening and impaired temperature compensation.

The above-described results suggest that the impaired temperature compensation is due to enhanced CK1a-mediated FRQ phosphorylation at high temperatures (Fig. 3). Thus, we predicted that impaired temperature compensation also can be caused by mutating some of the CK1a target sites on FRQ. FRQ protein is phosphorylated by CK1a and other kinases over 100 sites, and we have previously made different mutants in which multiple putative CK1a phosphorylation sites on FRQ are mutated (13, 14). M9 and M10 are two long-period mutants (29.8 and 32.1 h at 25°C, respectively) in which CK1a phosphorylation sites (FRQ 534-562) downstream of the FCD-2 domain of FRQ are mutated. Race tube assays showed that the increase of temperature from 21°C to 29°C resulted in a marked decrease of the period-lengthening effects in both mutants. The Q_10_ increased from approximately 1.14 for the wild type (wild-type *frq* complementation of *frq^KO^*) strain to approximately 1.39 and 1.33 for M9 and M10, respectively (Fig. 6A). These results confirmed that phosphorylation of FRQ mediated by CK1a is involved in the mechanism of temperature compensation.

How does the phosphorylation of the M9 and M10 region affect temperature compensation? *In vitro* kinase assay showed that the M9 and M10 region of FRQ (534-562) could be readily phosphorylated by CK1a, and the phosphorylation is relatively temperature insensitive (Fig. 3B). The M9 and M10 mutations were previously shown to weaken the FRQ-CK1a interaction (25). Thus, we tested whether the phosphorylation site mutation influences the structural conformation of FRQ, which, in turn, can affect the binding of FRQ with CK1a at different temperatures. We immunoprecipitated FRQ by endogenous CK1a in the M9 or M10 mutant and found that the relative amounts of the FRQ-CK1a complex in the M9 and M10 mutants were significantly higher at 29°C than those at 21°C. This result explains the partial loss of temperature compensation of these mutants (Fig. 6B). In contrast, FRQ became more stable at 29°C than at 21°C in the M9 and M10 mutants following either CHX treatment (Fig. 6C) or LD transition (Fig. S4B), further confirming that the temperature-dependent changes in FRQ stability were not responsible for their temperature compensation phenotypes. Together, these results strongly suggest that the CK1a-mediated FRQ phosphorylation at some sites is important for the maintenance of temperature compensation of circadian clock in *Neurospora*.

## DISCUSSION

Temperature compensation is a universal feature of all circadian clocks that allows organisms to adapt to different environmental conditions. Despite our current understanding of the eukaryotic clock mechanisms, how clocks achieve temperature compensation is unclear. Due to correlations between period length and stability of clock proteins in early studies, stabilities of FRQ and PER, the negative regulators of the clock in *Neurospora* and mammals, respectively, were previously proposed to regulate temperature compensation (30, 36, 59). Although FRQ stability can be correlated with circadian period in some clock mutants, we and others previously showed that FRQ phosphorylation rather than its stability determines circadian period length (25, 51). In this study, we showed that the impairment of FRQ-CK1a interaction, mutations of phosphorylation sites in FRQ, and a CK1a mutant with impaired FRQ phosphorylation all caused impaired temperature compensation. Moreover, FRQ stability was not affected in the wild-type strain at high temperatures at which temperature compensation was impaired, and FRQ actually became stabilized at high temperatures in some *frq* mutants with partial loss of temperature compensation. Thus, the temperature-compensated FRQ-CK1a interaction, but not FRQ stability, is the main biochemical process defining temperature compensation of the clock.

Unlike typical phosphorylation reactions in which the interaction between the substrate and the kinase is transient, the phosphorylation of FRQ by CK1a requires the formation of a stable FRQ-CK1a complex (24, 45, 46). We found that although CK1a level and activity are temperature insensitive, the relative FRQ-CK1a association is temperature insensitive in the wild-type strain below 30°C and is sensitive in different clock mutants with impaired temperature compensation. Multiple lines of evidence indicate that the maintenance of FRQ-CK1a interaction is the main mechanism that underlies temperature compensation of the clock in *Neurospora*. First, in the wild-type strain, the partial loss of temperature compensation above 30°C correlates with an increase of the FRQ-CK1a interaction. Second, in the *frq^7^* strain, which has partial loss of temperature compensation, the FRQ-CK1a interaction is enhanced at high temperature. Third, CK2 was previously shown to regulate temperature compensation in *Neurospora*, but unlike the *ck1a* mutant, the period of *ck2* mutants was temperature overcompensated (36). We confirmed the role of CK2 in temperature compensation and showed that the temperature compensation phenotypes of both *ckb* and FRQ CK2 phosphorylation site mutants are caused by a decrease of the relative amount of FRQ-CK1a association at high temperature. Fourth, and importantly, FRQ mutations that are specifically designed to impair the FRQ-CK1a interaction led to impaired temperature compensation. Finally, the mutations of FRQ phosphorylation sites mediated by CK1a also caused altered FRQ-CK1a interaction and temperature compensation phenotypes. Together, our results indicate that alterations in the FRQ-CK1a interaction explain both the loss of temperature compensation and temperature overcompensation phenotypes observed in the wild-type strain and different clock mutant strains. Our results indicate that the temperature-insensitive CK1a level, its kinase activity, and the temperature-compensated FRQ-CK1a interaction are responsible for the maintenance of similar circadian periods of the wild-type strain within the physiological temperature range (20 to 30°C). When the temperature is above 30°C, although CK1a protein levels and its kinase activity are not significantly changed, the structural conformation changes of FRQ cause enhanced FRQ-CK1a interaction, resulting in period shortening and impaired temperature compensation (Fig. 6D). In the temperature compensation-defective (*frq^7^*) and overcompensated (*ck2*) mutants, we predict that FRQ structure is differentially affected, resulting in an increase of FRQ-CK1a interaction in *frq^7^* and decreased FRQ-CK1a interaction in *ck2* mutant at high temperature.

We previously showed that the FRQ-CK1a interaction is the main process that determines circadian period length at room temperature due to its dual roles in both arms of the core circadian negative feedback loop that drive the circadian clock (25). The FRQ-CK1a interaction not only determines FRQ phosphorylation, which feeds back to regulate the FRQ-CK1a interaction and FRQ stability, but also determines the efficiency of the circadian negative feedback process by promoting the FRQ-dependent WC phosphorylation. Our results here further established the FRQ-CK1a interaction as the main determinant of the circadian period length at different temperatures. Both the loss of and overcompensated temperature compensation in different mutants are due to the temperature-sensitive FRQ-CK1 interaction.

Although reduction of CK1a expression level transcriptionally or posttranscriptionally was previously found to change period length in *Neurospora*, it caused no major temperature compensation defects (36, 63). A major difference between our work and those studies is that we directly perturbed the activity of CK1 (by inhibitors or mutation) or FRQ-CK1 interaction (by FRQ mutations/phosphorylation changes), whereas these previous studies only examined the effect of reducing the wild-type CK1 level. As we demonstrated here, mutations that impair FRQ phosphorylation and FRQ-CK1a interaction frequently make FRQ-CK1a interaction temperature sensitive, resulting in impaired temperature compensation. As indicated by the FRQ trypsin sensitivity change at high temperature and unchanged CK1a level (Fig. 3A and F), the temperature-sensitive FRQ-CK1a interaction is most likely caused by temperature-sensitive changes of FRQ protein structure independent of CK1a level. In contrast, despite the period changes due to reduction of CK1a level (36, 63), which should be caused by reduced FRQ-CK1a complex levels, both FRQ and CK1 proteins are still wild type in cells. As a result, the FRQ-CK1 interaction can remain temperature compensated, as in the wild-type strain.

It should be noted that in addition to CK1a, other kinases, such as CK2, can also phosphorylate FRQ and regulate circadian period length. Although their phosphorylation of FRQ may also influence FRQ-CK1 interaction and period length, it is also possible that these kinases phosphorylate at FRQ phosphorylation sites that regulate period length independent of FRQ-CK1a-mediated mechanism. Other mechanisms, such as the balance model, with different roles of kinases or a phosphoswitch-like model found in mammals may also contribute to circadian period determination and temperature compensation (30, 33, 36). Furthermore, additional processes that regulate WC complex activity can also regulate circadian period and, potentially, temperature compensation.

CK1-mediated clock protein phosphorylation is the most evolutionarily conserved process in the eukaryotic circadian mechanism. As it does in *Neurospora* with FRQ, CK1 forms a stable complex with PER proteins in *Drosophila* and mammals (12, 13, 18–23). Based on our findings in *Neurospora*, the PER-CK1 interaction may have a similar role in period determination in animal systems. In *Drosophila*, the DBT interaction domain of PER is critical for phosphorylation and its function in transcriptional suppression (64, 65). The PER-CK1 interaction domain appears to be conserved in mammals and is critical for PER2 function in mammalian cells (21, 23, 66, 67). In addition, phosphorylation of CLOCK-BMAL1 by CK1 in the complex was suggested to promote the dissociation of CLOCK-BMAL1 from DNA (20). Examination of the phosphorylation of mPER peptides by CKIδ *in vitro* previously led to the proposal that temperature-sensitive substrate and product binding of CKIδ underlies temperature-compensated phosphorylation and period length in the mammalian clock (35). The future mapping of the PER-CK1 interaction domain and mutations of PER that can disrupt the PER-CK1 interaction in an animal system should reveal the physiological roles of the PER-CK1 interaction in the mechanism of the animal circadian clocks.

## MATERIALS AND METHODS

### Strains, culture conditions, and race tube assays.

The 87-3 (*bd*, a) strain or an *frq* complementation strain (KAJ120) was used as the wild-type strain in this study. M9, M10, M19, *frq7*, *ckb^RIP^*, V320I, L323M, L488V, C490G, and Q494N strains were created previously (14, 25, 48). Constructs with the *qa-2* promoter driving expression of Myc.His.CK1a.hph were introduced into the 87-3 and *ckb^RIP^* strains at the *csr* locus by homologous recombination. Constructs with the *qa-2* promoter driving expression of Myc.His.CK1a.bar were introduced into the M19 strains at the *csr* locus by homologous recombination. Positive transformants were identified by Western blot analyses, and homokaryon strains were isolated by microconidial purification using 5-μm filters.

Liquid cultures were grown in minimal medium (1× Vogel’s, 2% glucose). When quinic acid (QA) was used to activate the *qa-2* promoter, liquid cultures were grown in 1 × 10^−2^ M QA (pH 5.8), 1× Vogel’s, 0.1% glucose, and 0.17% arginine. For rhythmic experiments, *Neurospora* was cultured in petri dishes in liquid medium for 2 days. The *Neurospora* mats were cut into discs, transferred into medium-containing flasks, and harvested at the indicated time points.

The medium for race tube assays contained 1× Vogel’s salts, 0.1% or 0.03% glucose, 0.17% arginine, 50 ng/ml biotin, and 1.5% agar with or without QA. After entrainment for 24 h in constant light, race tubes were transferred to constant dark and the growth front marked every 24 h. The position of the conidiation band was used to determine the time of banding (circadian period of the *Neurospora*) relative to the growth front marks. Q_10_ value was calculated by the equation Q_10_ = (τ1/τ2)^10/(T2‐T1)^, where τ1 and τ2 are the periods at temperature T1 and T2, respectively.

### Creation of the *ck1a* knock-in strains.

To create the *ck1a* knock-in strain, a wild-type knock-in cassette with a hygromycin resistance gene (*hph*) was inserted downstream of the *ck1a* 3′ untranslated region (UTR). The cassette was then transformed into the *ku70^RIP^* strain to select for *hph*-resistant transformants. The homokaryotic strains were obtained by microconidial purification, crossing with a wild-type (301-6) strain, and confirmed by DNA sequencing (24, 68). Using this method, we created the *ck1a^WT^* and *ck1a^H123Y^* mutant knock-in strains at the *ck1a* endogenous locus.

### Protein analyses.

Protein extraction, quantification, and Western blot analyses were performed as previously described (69–71). Briefly, tissue was ground in liquid nitrogen with a mortar and pestle and suspended in ice-cold extraction buffer (50 mM HEPES [pH 7.4], 137 mM NaCl, 10% glycerol) with protease inhibitors (1 μg/ml pepstatin A, 1 μg/ml leupeptin, and 1 mM phenylmethylsulfonyl fluoride). After centrifugation, protein concentration was measured using a protein assay kit (Bio-Rad). For Western blot analyses, equal amounts of total protein (40 μg) were loaded in each lane of 7.5% or 10% SDS-PAGE gels containing a ratio of 37.5:1 acrylamide to bisacrylamide. After electrophoresis, proteins were transferred onto polyvinylidene difluoride (PVDF) membranes, and Western blot analyses were performed. Western blot signals were detected by X-ray films and scanned for quantification.

### Immunoprecipitation and Flag pulldown analyses.

Immunoprecipitation analyses were performed as previously described (69). Briefly, *Neurospora* proteins were extracted as described above. For each immunoprecipitation reaction, 2 mg protein and 1.5 μl c-Myc antibody, 1 μl WC-2 antibody (72), or 1 μl CK1a antibody were used. After incubation with antibody for 3 h, 40 μl GammaBind G Sepharose beads (GE Healthcare) was added, and samples were incubated for 1 h. For the Flag pulldown assay, 4 mg purified Flag-CK1a fusion protein and 2 μl Flag antibody were incubated with 2 mg *Neurospora* extracts. After incubation with antibody for 3 h, 40 protein GammaBind G Sepharose beads were added, and samples were incubated for 1 h. Immunoprecipitated proteins were washed three times using extraction buffer before Western blot analysis. IP experiments were performed using cultures harvested in constant light. Quantification of relative FRQ-CK1a or FRQ-WC-2 interaction levels is based on the ratio of IP to input and normalized with CK1a or WC-2 levels, respectively. All FRQ isoforms (hyperphosphorylated and hypophosphorylated FRQ) and all CK1 isoforms (the endogenous CK1a has two spliced isoforms, and quinic acid-induced CK1a has three isoforms) were used for quantifications.

### *In vitro* kinase assays.

For the *in vitro* phosphorylation analyses, His-CK1a, His-CK2a, GST-FRQ (534-562), GST-FRQ (425-683), GST-FRQ (972-990), and GST-FRQ (972-990)-M19 fusion proteins were purified from Escherichia coli BL21(DE3) cells (49, 68). His-CK1a or His-CK2a fusion protein (0.5μg) was incubated with GST-FRQ (534-562), GST-FRQ (425-683), GST-FRQ (972-990), or GST-FRQ (972-990)-M19 fusion protein (5 μg) in the reaction buffer (total, 20 μl) containing 25 mM HEPES (pH 7.9), 10 mM MgCl_2_, 2 mM MnCl_2_, 25 μM ATP, and 2 μCi of [γ-^32^P]ATP (49). The reaction mixture was incubated for 15, 30, and 60 min at temperatures ranging from 20°C to 35°C, and the proteins were boiled in 1× SDS loading buffer and separated by SDS-PAGE. After electrophoresis, the gel was dried and the level of phosphorylation was assessed by autoradiography. Each experiment was independently performed at least three times.

### Protein stability assays.

To examine FRQ protein stability, discs made from 2-day-old mycelial mats of individual strains were grown in liquid medium for 1 day under constant light conditions. Cultures were grown in constant light for 1 day prior to the addition of CHX to a final concentration of 10 μg/ml or were transferred into constant darkness and harvested at the indicated time.

### Trypsin digestion assays.

For the trypsin sensitivity assay, protein extracts were diluted to a protein concentration of 2.5 μg/μl. A 100-μl aliquot was treated with trypsin (final concentration, 1 μg/ml) at 25 °C. A 20-μl sample was taken from the reaction mixture at each time point (0, 5, 15, and 30 min) after addition of trypsin. Protein samples were mixed with protein loading buffer and resolved by SDS-PAGE. To compare trypsin sensitivity of FRQ from different strains, experiments were performed side by side, and the protein samples were transferred to the same membrane for Western blot analysis.

### Quantification and statistical analyses.

Quantification of Western blot data was performed using Image J software (73). All studies were performed on at least three independent experiments. Error bars are standard deviations for immunoprecipitation assays and standard errors of the means for race tube assays. Statistical significance was determined by Student’s *t* test.

### Data and material availability.

Materials are available from the corresponding authors upon reasonable request.
