# Supplementary figures and images for: FRQ-CK1 Interaction Underlies Temperature Compensation of the Neurospora Circadian Clock
Source: mBio. 2021 Jun 29;12(3):e01425-21. doi: 10.1128/mBio.01425-21 (PMC8263009; doi:10.1128/mBio.01425-21)

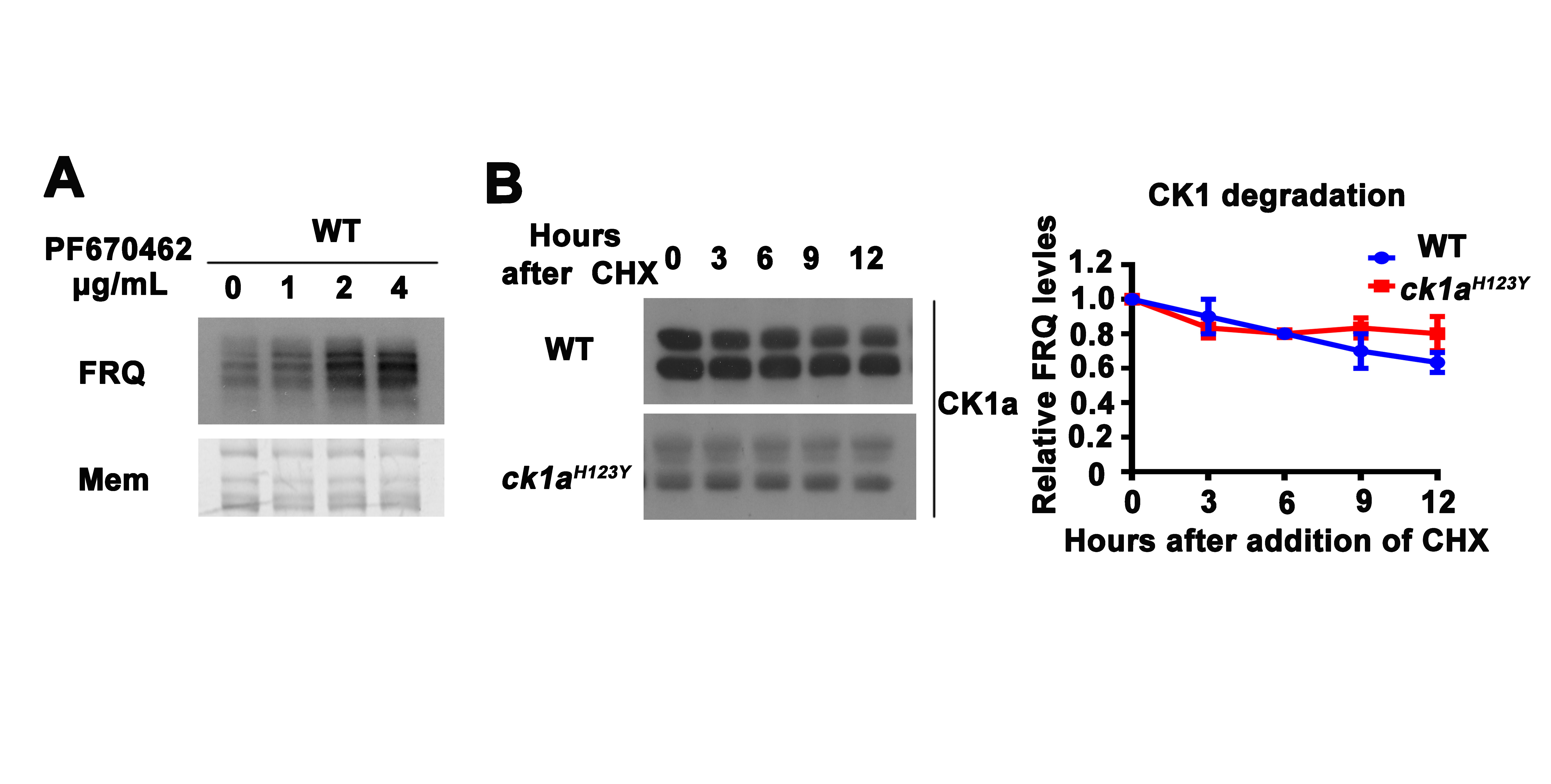

Supplement: FIG S1 [file mbio.01425-21-sf001.tif]

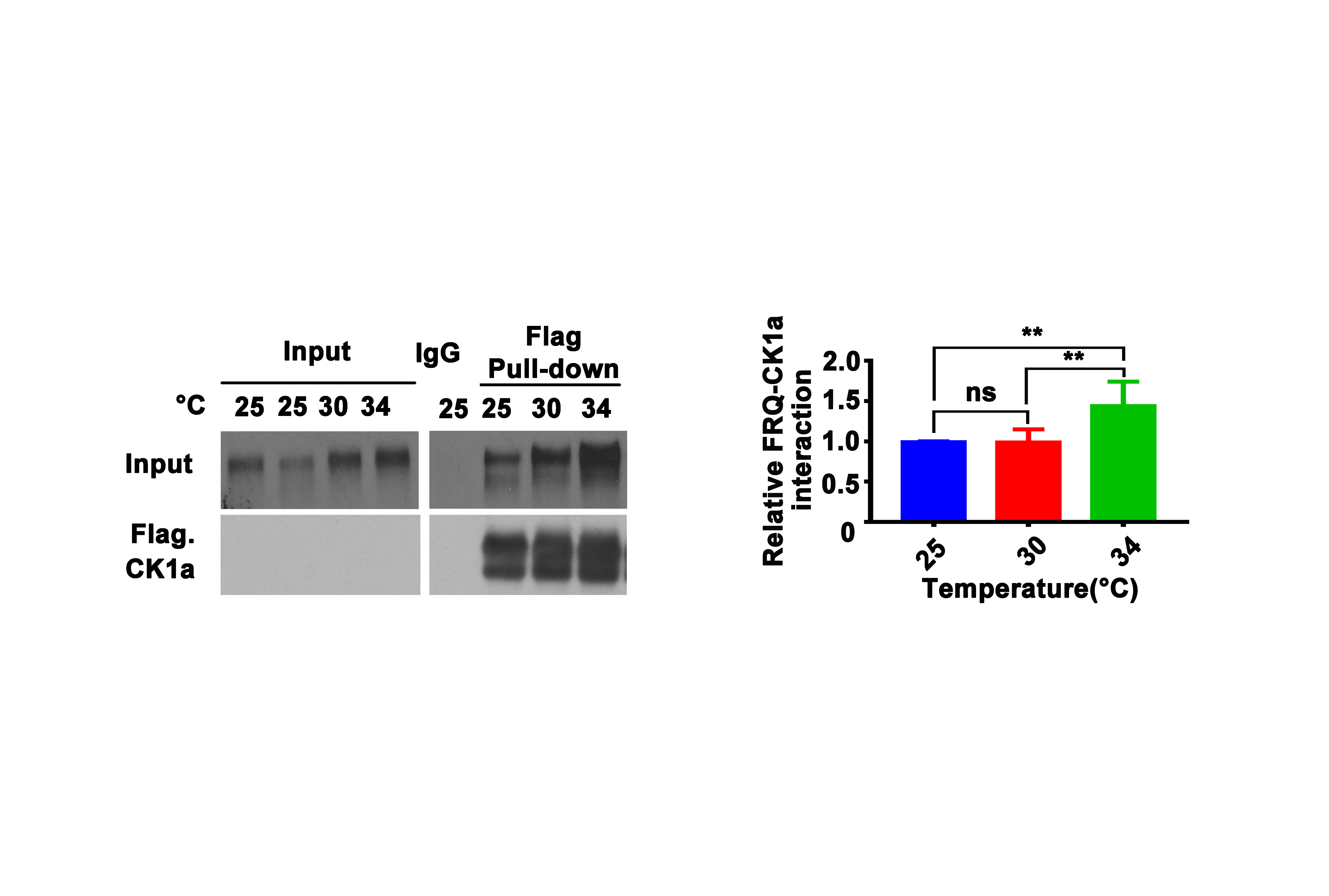

Supplement: FIG S2 [file mbio.01425-21-sf002.tif]

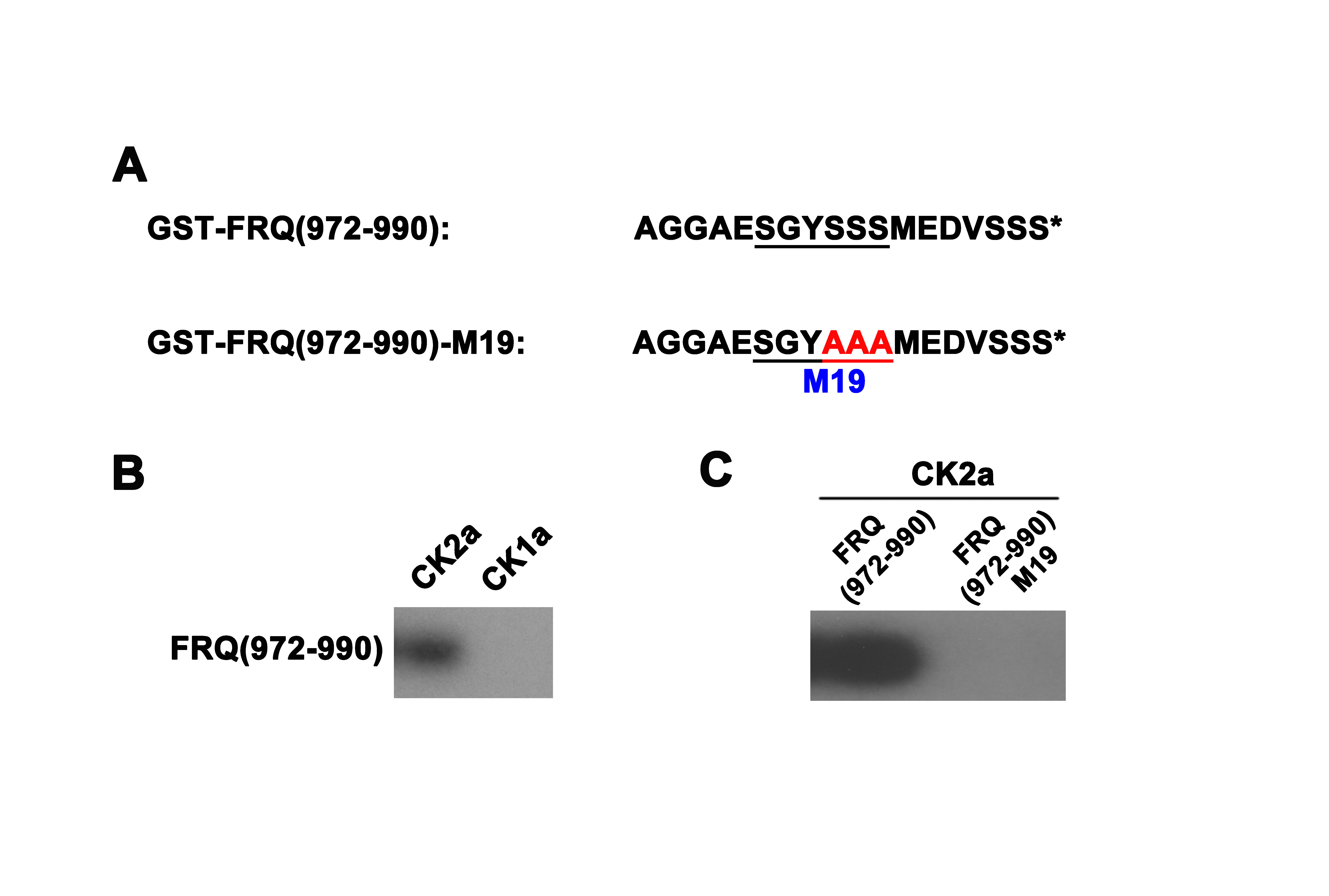

Supplement: FIG S3 [file mbio.01425-21-sf003.tif]
